# The pancreatic β cell: recent insights from human genetics

**DOI:** 10.1016/j.tem.2014.05.001

**Published:** 2014-08

**Authors:** Soren K. Thomsen, Anna L. Gloyn

**Affiliations:** 1 Oxford Centre for Diabetes, Endocrinology, and Metabolism, University of Oxford, Headington, OX3 7LE, UK; 2 Oxford National Institute for Health Research (NIHR) Biomedical Research Centre, Churchill Hospital, Headington, OX3 7LE, UK

**Keywords:** pancreatic β cell, human genetics, diabetes, GWAS

## Abstract

Diabetes mellitus is a metabolic disease characterised by relative or absolute pancreatic β cell dysfunction. Genetic variants implicated in disease risk can be identified by studying affected individuals. To understand the mechanisms driving genetic associations, variants must be translated through causative transcripts to biological insights. Studies into the genetic basis of Mendelian forms of diabetes have successfully identified genes involved in both β cell function and pancreatic development. For type 2 diabetes (T2D), genome-wide association studies (GWASs) are uncovering an ever-increasing number of susceptibility variants that exert their effect through β cell dysfunction, but translation to mechanistic understanding has in most cases been slow. Improved annotations of the islet genome and advances in whole-genome and -exome sequencing (WHS and WES) have facilitated recent progress.

## Highlights

• Genes involved in pancreatic development are implicated in Mendelian diabetes.
• Studying the genetic basis of diabetes has delivered insights into β cell biology.
• Most genetic associations influence diabetes risk through defects in β cell function.
• Sequencing-based methods and improved genomic annotations have facilitated advances.

## Interrogating the genetic basis of diabetes

Over the past decades diabetes mellitus has been increasing dramatically in prevalence and is predicted to affect 592 million worldwide by 2035 [1]. Auto-immune forms currently make up 5–10% of all cases, and the remainder predominantly comprises T2D. Changes in diet and lifestyle are key drivers behind the T2D pandemic, but heritable factors are known to contribute to a significant proportion of the phenotypic variance 2, 3. Studies into the genetic susceptibility of T2D have supported the notion that the disease develops due to an inability of the pancreatic β cell to compensate for increasing peripheral insulin resistance 4, 5. The primary function of the β cell is in metabolic homeostasis, achieved through a coupling of ambient glucose levels with insulin secretion. In T2D and related, rare forms of Mendelian diabetes, relative or absolute β cell dysfunction leads to hyperglycaemia, causing severe complications over time.

The study of human genetics provides a powerful tool to understand the mechanisms underlying β cell dysfunction in humans. Studies of individuals or populations with diabetes can be used to identify genetic variants (see Glossary) that either cause or increase susceptibility to disease. For neonatal diabetes mellitus (NDM) and maturity onset diabetes of the young (MODY), referred to collectively as Mendelian diabetes, the variants are rare and highly penetrant. Regions containing such variation were traditionally identified by linkage analysis and positional cloning (Box 1), but technological advances have recently resulted in WES and WGS becoming more common. For T2D, which is a multifactorial disease, the susceptibility variants have smaller effect sizes and can become more common due to reduced purifying selection. The majority of variants established to date have been identified through common variant association studies (CVASs), a type of GWAS targeting common variants (Box 1) [6]. The associations, however, have been found insufficient to account for measures of T2D heritability. To address this, rare variant association studies (RVASs) have been increasingly used to capture lower-frequency variants of moderate effect sizes (Box 1). The findings from studies on the genetics of both Mendelian and complex types of diabetes provide biological insights that have the potential to be translated directly into clinically relevant disease mechanisms. This advantage to human genetics is particularly evident in cases where rodent models have been found to inadequately recapitulate human phenotypes 7, 8, 9.

Box 1 **Identifying genomic regions implicated in diabetes risk**

*Linkage analysis and positional cloning*

For highly penetrant mutations causing Mendelian diabetes, positional cloning has been an invaluable tool to identify genes implicated in disease pathogenesis. The principle is based on the tendency of genetic elements in close proximity to be coinherited. By mapping disease-causing variants relative to cosegregating genetic markers (DNA sequences of known position that show variation in sequence or length between individuals) the approximate position in the genome can be established. Typically, this approach will identify a candidate gene region covering millions of basepairs. Following this, several overlapping clones covering the region can be generated and the causative gene identified.

*GWAS, RVAS, and CVAS studies*

For complex forms of diabetes, numerous genes with small effect sizes and low penetrance contribute to disease susceptibility. Thus, rather than using linkage-based approaches, GWASs are applied to large samples of individuals with and without diabetes to establish statistical evidence for association of particular variants with the disease. The ratio between the frequency of an allele in cases compared with controls is referred to as the odds ratio (OR), a measure of the effect size of a variant. An OR of 1 indicates no effect, whereas an OR significantly different from 1 shows evidence of either a protective (OR <1) or harmful effect (OR >1). GWASs that target common SNPs [minor allele frequency (MAF) >5%)] using microarray technology are referred to in this review as common variant association studies (CVASs). By contrast, studies that target low-frequency (0.5% > MAF > 5%) and rare variants (MAF < 0.5%) by either sequencing-based approaches or microarrays designed to capture such SNPs are referred to as rare variant association studies (RVASs). Although often simply called exome-sequencing studies, we refrain from using this terminology to avoid mixing up statistical and genotyping methodologies [6].

**Figure 1:**
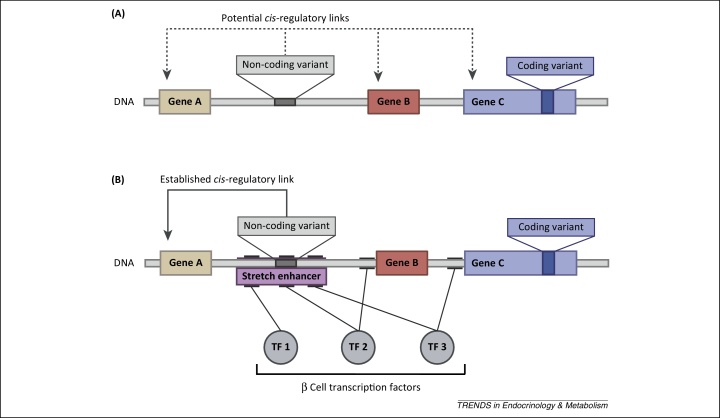
The mechanisms underlying disease-associated genetic variants. **(A)** Location of a non-coding and a coding genetic variant relative to three genes: A, B, and C. The coding variant is likely to exert its influence on disease pathogenesis through an effect on the function of gene C. Several different mechanisms, including amino acid substitution and protein truncation, could be driving the association. The non-coding variant, on the contrary, could be acting through any of the three nearby genes, as shown by the dotted arrows indicating potential *cis*-regulatory links. The variant could, for example, affect either expression levels or splicing patterns, but identifying the causative transcript can be challenging. **(B)** The schematic depicted in panel A with additional functional annotations (Box 2 for details). The binding sites of three β cell specific transcription factors (TF 1, 2 and 3) are shown by lines connected to the respective proteins. Three binding sites cluster to form a stretch enhancer, which is likely to have islet-specific *cis*-regulatory activity (see the section ‘Understanding the impact of regulatory variants on islet gene function’). The non-coding variant is seen to overlap with the binding site of TF2 within the stretch enhancer, and could therefore plausibly act through disruption of TF2 binding. The solid arrow shows an established *cis*-regulatory link between the variant and gene 1. This relationship could be supported by different types of functional annotation, including a correlation between the non-coding variant and expression of gene 1 (established by *cis*-eQTL studies) or a physical interaction between the enhancer and the promoter region of gene 1 (demonstrated by chromatin-conformation capture, 3C) (Box 2). With such annotations at hand, identifying the causative transcript at a disease-associated locus is a more tractable problem.

Despite advances in identifying genetic variants that increase diabetes susceptibility, the translation of association signals into molecular mechanisms has in many cases been slow. Exceptions include regions where biologically relevant genes harbour coding variants [10]. Many signals, however, are located in intergenic regions, and assigning a causative transcript to a particular variant presents a significant challenge (Figure 1A). Recent progress in structurally and functionally annotating the islet genome has provided a context in which to understand the effects of non-coding variants, and has in some cases facilitated translation into biological mechanisms (Figure 1B) 11, 12, 13, 14.

In this review we first describe the current state of genomic annotation in islets, focusing on the biological impact of this work. Second, we discuss new insights into β cell biology from variants causing or increasing susceptibility to T2D and related forms of Mendelian diabetes. Where relevant we highlight the use of islet genome annotation to interrogate variation, a strategy that will become increasingly important in understanding how non-coding variants impact on the molecular machinery of the β cell.

## Understanding the impact of regulatory variants on islet gene function

Genomic annotation is the process of assigning structural and functional meaning, such as gene structure and chromatin modifications, to specific sequences in the genome (Box 2). Many of these features are dynamically regulated, and therefore vary according to developmental stage, metabolic state, or tissue type. The ENCODE project has made significant progress in cataloguing genomic features (including various histone modifications and transcription factor binding sites) for a large number of cell types, but does not currently include either pancreatic islets or β cells. To obtain information that is directly relevant to our understanding of those diabetes risk variants that exert their effect through β cell dysfunction, several groups have recently addressed this issue. In most of these studies, islets of Langerhans have been used as a proxy for β cells, which make up 65–80% of the human islets but are hard to isolate as a pure fraction. Some have focused on identifying transcription factor binding sites or regions with specific states of chromatin 13, 14, 15, 16, 17, 18, 19.

Box 2 **Annotating the genome**

Cataloguing transcription factor binding sites and epigenetic features, such as histone modifications, can shed light on the function of regulatory genomic regions. Evidence for this has been provided by recent studies in lymphoblastoid cell lines which have demonstrated that non-coding SNPs, at least to some extent, exert their *cis*-regulatory effects through disruption of transcription factor binding (Figure 1B) 68, 69, 70. The changes in occupancy in turn alter the chromatin landscape both locally and at the promoter region of nearby genes, thereby influencing transcriptional efficiency.

*Chromatin states and transcription factor networks*

To functionally annotate genomic features, microarray analysis or WGS is applied to samples of DNA that are enriched or pretreated in different ways. One of the most commonly used methods is chromatin immunoprecipitation (ChIP) followed by microarray analysis (ChIP-chip) or sequencing (ChIP-seq). ChIP makes use of antibody-based purification to select genomic elements that are associated with either specific transcription factors or modified histones. Alternatively, isolation and identification of DNA fragments not associated with proteins can identify regions of open chromatin through so-called formaldehyde-assisted isolation of regulatory elements (FAIRE). Further, to map out DNA methylation patterns, approaches based on bisulfite conversion can be used to distinguish unmethylated from methylated cytosines.

*Variant-transcript regulatory links*

A higher level of functional information is present in the relationship between two distinct genomic elements. An important example is the statistical correlation between a variant and the expression of a particular gene. Such variants, so-called expression quantitative trait loci (eQTLs), can be found by combining transcriptomic and genomic datasets from islets, but require large sample sizes to obtain adequate statistical power. Once identified, overlapping GWAS association signals with *cis*-eQTLs (local eQTLs) can in principle provide strong evidence in support of a specific causative transcript (Figure 1). Large-scale studies are coming out, but early studies have already demonstrated that particular expected *cis*-eQTLs can be identified at a relaxed *P* value threshold [24]. Finally, variant-transcript links can also be identified by exploiting the fact that enhancers tend to interact with the promoter regions of target genes. By chromatin-conformation capture (3C)-based approaches it is possible to identify genomic elements that are in close spatial proximity owing to folding and looping of chromatin.

Other studies have performed transcriptomic analyses to provide a wealth of information on the expression of both protein-coding genes and non-coding RNAs, thereby expanding the classes of structural elements that can be interrogated when considering the impact of genetic variants 20, 21, 22, 23. Such datasets have also been integrated with genomic data to enable genome-wide testing for variants that affect transcript levels, so-called expression quantitative trait loci (eQTL) (Box 2) 23, 24. Early studies have been limited by insufficient statistical power owing to relatively small sample sizes, but large-scale eQTL efforts in islets are currently underway and are anticipated to provide an important layer of regulatory information to the islet genome.

One of the key lessons emerging from the genomic annotations has been an increased understanding of how islet-specific genes are regulated. Several studies have made observations which indicate that genes involved in β cell identity and function tend to be regulated by clusters of transcription factors rather than by orphan enhancers 13, 14, 17. The clusters form stretch- or super-enhancers that may act as molecular runways for tissue-specific transcription factors 17, 25, 26 (Figure 1B). Importantly, the islet stretch enhancers are found to be enriched for variants associated with susceptibility to diabetes and related metabolic traits 13, 17.

## From association signal to molecular mechanism

### Mendelian diabetes

**Figure 2:**
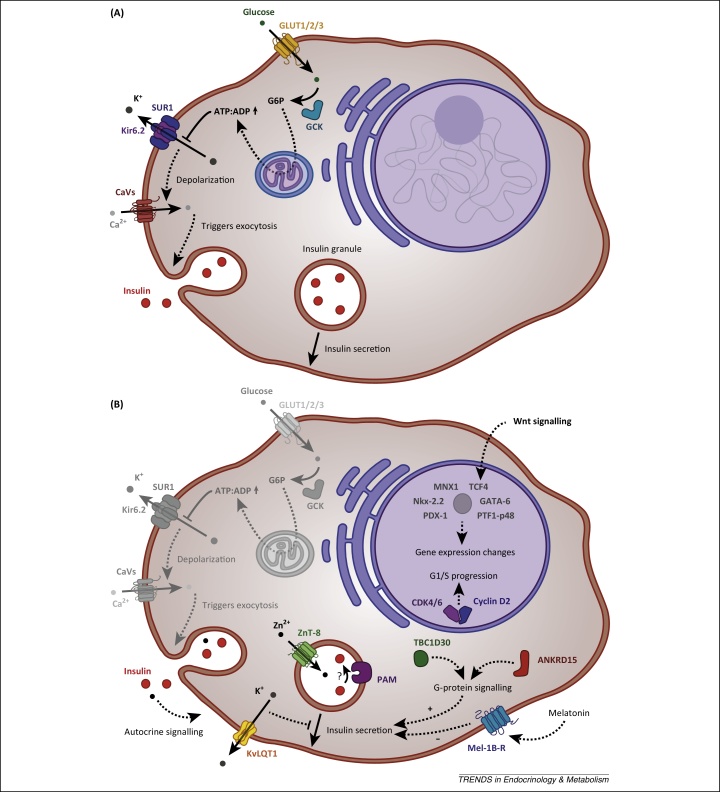
Schematic representation of the pancreatic β cell and the location of key components implicated by human genetics in diabetes pathogenesis. Solid arrows indicate direct mechanisms of action and dotted arrows indicate mechanisms with intermediate events not displayed in the schematic. **(A)** Consensus model of glucose-stimulated insulin secretion (GSIS), including several key proteins involved in Mendelian forms of diabetes. Proteins depicted: GCK (encoded by *GCK*), GLUT1/2/3 (*SLC2A1/2/3*), insulin (*INS*), Kir6.2 (*KCNJ11*), SUR1 (*ABCC8*), and voltage-gated Ca^2+^ channels (CaVs), which represent both P/Q type and L-type channels ([71] for details). Briefly, glucose enters the cell through one of the glucose transporters GLUT1/2/3 and is phosphorylated by GCK to form glucose-6-phosphate (G6P). In the mitochondria, aerobic metabolism of glycolytic products results in the generation of ATP. Changes in the ATP:ADP ratio inactivates the ATP-sensitive potassium channel K_ATP_, which consists of four SUR1 and four Kir6.2 subunits. Closure of K_ATP_ in turn causes depolarization of the membrane opening voltage-gated Ca^2+^ channels. Finally, the influx of Ca^2+^ triggers exocytosis of insulin granules. **(B)** Location and proposed role in β cell function of proteins encoded by genes discussed in this review. Proteins depicted: ANKRD15 (encoded by *KANK1*), CDK4/6 (*CDK4 and CDK6*), cyclin D2 (*CCND2*), KvLQT1 (KCNQ1), Mel-1B (*MTNR1B*), PAM (*PAM*), TBC1D30 (*TBC1D30*), ZnT-8 (*SLC30A8*), and the transcription factors GATA-6 (*GATA6*), MNX1 (*MNX1*), Nkx-2.2 (*NKX2-2*), PDX-1 (*PDX1*), PTF1-p48 (*PTF1A*), and TCF4 (*TCF7L2*). See main text for details of individual genes.

Mendelian forms of diabetes encompass a group of diseases, including both MODY and NDM, which show a Mendelian pattern of inheritance. Because of the clear genotype–phenotype correlation they constitute an invaluable tool for understanding how the disruption of a particular gene can impact on molecular and physiological pathways in humans. Studies into the genetics of Mendelian diabetes have been remarkably successful in identifying genes implicated in pathogenesis, including both components of the insulin secretory pathways (e.g., *ABCC8, GCK, INS, KCNJ11*) (Figure 2A) and developmentally important transcription factors (e.g., *HNF1A, HNF4A, PDX1, PTF1A*) [27]. In some instances the insights arising from these studies have enabled substantial therapeutic advances, most notably in the case of sulfonylurea treatment for patients with mutations in genes encoding subunits of the K_ATP_ channel (*ABCC8* and *KCNJ11*) [28].

The genetics of Mendelian diabetes is continuing to provide insights into β cell function, facilitated by advances in sequencing-based approaches, such as WES and WGS, and in genomic annotation. WES offers the possibility to discover coding, disease-causing mutations in an untargeted manner, as recently demonstrated by a study investigating the genetic aetiology of pancreatic agenesis [8]. This rare condition, which clinically manifests itself through both diabetes and exocrine pancreatic insufficiency, is caused by complete or partial failure of the pancreas to develop. Lango Allen and colleagues performed WES on a subset of probands from a cohort of individuals with unexplained pancreatic agenesis. The authors used a common strategy of filtering against synonymous and known variation to enrich for likely pathogenic variants, and this identified heterozygous *de novo* mutations in the *GATA6* (GATA-binding factor 6) gene. Targeted sequencing of *GATA6* in further subjects revealed additional non-synonymous mutations in over half the individuals. Mutations in *GATA6* have subsequently been shown in follow-up studies to cause a spectrum of diabetic phenotypes, ranging from pancreatic agenesis to adult-onset diabetes with no overt exocrine insufficiency 29, 30. This incomplete penetrance of *GATA6*-inactivating mutations has also been noted by Bonnefond *et al.*, who showed that cardiac malformation is a more consistent phenotype of mutation carriers [31]. The variability in clinical presentation presumably reflects both mutational burden as well as genetic background. Overall, these studies have established that *GATA6* haploinsuffiency is a major cause of syndromic pancreatic agenesis and point to a crucial role for GATA6 in pancreatic development. Furthermore, this discovery highlights the value of using human genetics to understand gene-dosage relationships for dominant variants because heterozygous *Gata6* knockout mice show no apparent phenotype [8].

Unlike exome-based approaches, WGS can be used to identify both coding and regulatory variants. In non-coding regions, however, predicted disruption of protein function cannot be used as an indicator of pathogenicity. A challenge for WGS-based studies into Mendelian diseases is therefore related to the filtering of incidental variation from disease-causing mutations, and particularly in cases where linkage information is limited. A study into the genetic basis of patients with non-syndromic pancreatic agenesis (no extra-pancreatic features) explored the potential in combining WGS with spatially and temporally relevant genomic annotations to identify a subset of functionally active regulatory variants [11]. The study focused on homozygous mutations, consistent with the observation that close to half of the studied individuals were to born to consanguineous parents 8, 11. Failing to identify any plausible coding variants, the authors extended their search to developmentally active enhancer regions. Such an annotated map was obtained by defining enhancers bound by multiple transcription factors in pancreatic endoderm derived from human embryonic stem cells. In this way, a putative stretch enhancer downstream of *PTF1A* (pancreas transcription factor 1) was found to contain homozygous mutations in 9 of 12 individuals with isolated pancreatic agenesis. Compelling genetic and functional evidence established the pathogenicity of the variants, which all seem to abolish enhancer activity through disruption of transcription factor binding. Recessive coding mutations in *PTF1A* have previously been implicated in Mendelian diabetes, but are in all these cases associated with neurological features 32, 33. Interestingly, the distal enhancer found to harbour mutations causing isolated pancreatic agenesis does not have active marks in any ENCODE-annotated tissue type, suggesting that tissue-specific regulation can account for the absence of extra-pancreatic features [11].

The observation that some mouse models (e.g., *GATA6, HNF1A*) show poor phenotypic correlation with humans questions the degree to which mice can provide relevant insights into human pancreatic development 7, 8, 34. To address this issue more systematically, Flanagan *et al.* screened for homozygous mutations in individuals with NDM born to consanguineous parents [9]. The study focused on 29 genes known to be important regulators of mouse pancreatic development. In total, seven of the genes were found to be affected in the studied collection, including *NKX2-2* (NK2 homeobox protein 2) and *MNX-1* (motor neuron and pancreas homeobox 1), which had not previously been associated with NDM. Comparison between the clinical features of the subjects (both pancreatic and extra-pancreatic) and the corresponding phenotypes of homozygous-null mouse models revealed pronounced similarities across all seven genes. Thus, it seems that the phenotypic correlation between humans and mouse models is generally high for biallelic inactivations.

Overall, the genetics of rare Mendelian forms of diabetes have provided a wealth of information into both the function of β cells and more recently also pancreatic development (Figure 2). The insights generated by these and other studies enhance our understanding of the transcriptional networks that regulate human pancreatic development 35, 36. Integration of genetics and functional genomics has been established as a powerful tool to interrogate regulatory variation, but the proportion of non-coding variants causing Mendelian diseases is still uncertain [6].

### Type 2 diabetes

**Figure 3:**
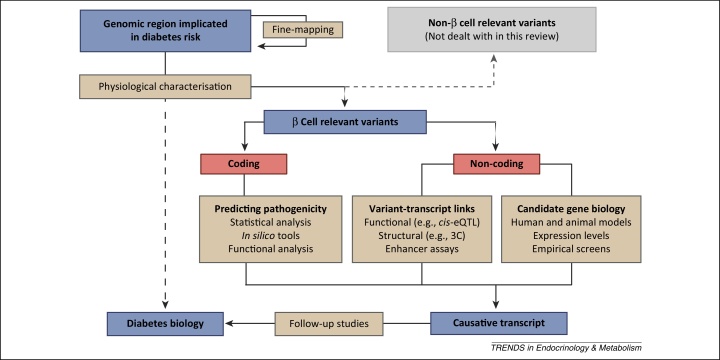
From association signal, through causative transcript, to biology. Beige boxes contain key experimental procedures; blue boxes indicate the entity of focus along the process of translating genetic associations to biology; and red boxes are divisions of such entities. As summarised in Box 1, human genetics can identify genomic regions implicated in diabetes risk. Subsequently, fine-mapping approaches can refine the genetic associations and identify the causative variant. Physiological characterisation of individuals carrying those risk alleles can in turn reveal effects indicative of the tissue(s) affected, but only indirect evidence for the underlying mechanisms (dotted line). Instead, identifying the transcripts driving the association of β cell relevant variants provides more direct insight into disease biology. For coding variants this can be achieved by prediction or validation of pathogenicity, whereas for non-coding signals consideration of prior candidate gene biology for nearby genes or variant transcript links can prioritize regional transcripts. Once a causative transcript has been identified, several different approaches, including human models, animal models, and functional studies, can be used to interrogate the function of the protein and ascertain its role in disease biology. Abbreviations: 3C, chromatin conformation capture; *cis*-eQTL, *cis*-expression quantitative trait loci (Box 2).

In contrast to Mendelian forms of diabetes, the genetic contribution to T2D is obscured by environmental influences and the relatively low penetrance of susceptibility variants. As a consequence, genetic variants are identified through association studies by establishing statistical evidence for overrepresentation in cases compared with controls (Box 1). Once identified at a stringent genome-wide significance threshold, variants must be linked to causative transcripts, which can be interrogated for biological function (Figure 3). A few of the 77 susceptibility regions previously found to be associated with T2D contain a non-synonymous variant in the coding region of a gene 4, 5, 37. In such cases (e.g., *KCNJ11*, *SLC30A8*, and *GCKR*), identifying the causative transcript is relatively straightforward and can be immediately followed up with functional studies and animal models [10]. For the remaining loci, however, the variants reside in non-coding regions of the genome, making it a challenge to establish a causal link between the signal and a nearby gene (Figure 1). We discuss here progress concerning variants from across the frequency spectrum.

### Common variants

Most of the established T2D-associated loci are common variants that have been identified through CVASs 4, 5, 37. Physiological characterisations of carriers have demonstrated that, although T2D is etiologically heterogeneous, the majority of these association signals appear to act through β cell dysfunction 4, 38, 39. Thus, inferring the causative transcripts from risk variants holds the promise to identify genes that are important for β cell function.

In the case of the *TCF7L2* (transcription factor 7-like 2, T cell specific, HMG-box) locus, improvements in islet genomic annotations have provided new insights into the mechanism of an intronic risk variant 13, 14. The association signal has been the focus of particular interest because of its relatively large effect size [odds ratio (OR) = 1.4 in the CEU (Central European) cohort], the highest of any common variant in Caucasian populations [5]. Despite the high OR, functional studies have shown inconsistent effects of *TCF7L2* levels on β cell function, and the primary tissues through which the effect is expressed has remained controversial 40, 41, 42, 43, 44. Genomic annotations, however, have provided several lines of evidence supporting islet-specific enhancer activity. First, Gaulton *et al.* were able to show that the *TCF7L2* association signal is located in an islet-selective region of open chromatin, indicative of regulatory activity [14]. The risk allele is itself associated with a more accessible state of chromatin and increased enhancer activity in β cell lines 14, 18. Further, it has been demonstrated by a comprehensive analysis of transcription factor binding sites in islets that the region containing the lead single-nucleotide polymorphism (SNP) is a stretch enhancer, bound by three key islet transcription factors [13]. Together, these observations provide strong evidence in favour of a β cell driven effect on T2D susceptibility (Figure 2B).

A different type of regulatory relationship has been described for the intronic variants at the *KCNQ1* (potassium voltage-gated channel, KQT-like subfamily, member 1) locus. The region contains both multiple independent association signals and biologically plausible transcripts, including *KCNQ1* (Figure 2B). Consistent with the imprinted status of the locus, it is one of only two T2D loci to display a strong parent-of-origin effect 45, 46. Physiological characterisation of individuals with loss-of-function mutations in *KCNQ1* has demonstrated increased insulin secretion, highlighting *KCNQ1* as a likely causative transcript [47]. This is reinforced by a study investigating the imprinting status of regional transcripts in foetal and adult islets [12]. Evidence of monoallelic expression was observed for *KCNQ1* in the foetal but not in the adult state, arguing that any potential effect on T2D must be developmental. Thus, *KCNQ1* is emerging as a strong candidate at the locus, although the possibility of effects on other regional transcripts, such as *CDKN1C* (cyclin-dependent kinase inhibitor 1C; encoding p27^KIP2^), cannot be excluded [19].

### Low-frequency and rare variants

Despite the large number of common variants having been discovered by CVASs, the combined population-attributable risk accounts for only a small fraction of the total heritability of T2D (Box 3) 4, 5. This has prompted speculations that rare and low-frequency variants of moderate effect sizes could explain a significant proportion of the ‘missing heritability’ [48]. Early RVASs have addressed this conundrum directly by targeting either specific regions already established by CVAS, or by examining the whole exome or genome in an unbiased fashion 49, 50, 51, 52.

Box 3 **Missing heritability in T2D**

Common variants identified to date together account for less than 10% of the variance in diabetes susceptibility estimated by twin and segregation studies 5, 48. To account for this ‘missing heritability’ several potential sources have been proposed, including gene–environment interactions, structural variants not captured by existing GWASs, rare variants of moderately high effect size, incorrect estimates of heritability, and inappropriate study design. Rare variants in particular have attracted attention recently and the focuses of many GWASs have shifted from CVAS towards RVAS. The notion of rare variants contributing to disease susceptibility is supported by resequencing of the diabetes susceptibility genes *KCNJ11* and *HHEX*, which revealed an unexpectedly large number of rare variants arising because of recent explosive population growth [72]. Conversely, modelling of the distribution of common variants has shown that a very large number of such variants may be associated with T2D [5]. These would explain a majority of the heritability but require unfeasibly large populations for genome-wide significance owing to progressively smaller effect sizes. Supporting this, Lohmueller and colleagues combined simulations with sequencing of a limited number of individuals, and concluded that their observations were inconsistent with a model where rare variants in a modest number of genes explain the majority of diabetes susceptibility [73]. However, recent studies with larger samples sizes have been able to establish exome-wide levels of statistical significance for rare variants at both new and previously established T2D loci (main text for details). As for common variants, it appears that the limited number of rare variants identified so far primarily affect β cell function. Overall, despite their relatively high ORs, the population-attributable risks remain low because of their low frequencies, and it remains to be seen whether rare variants contribute significantly to T2D heritability.

An initial study of this kind focused on *MTNR1B* (melatonin receptor 1B), a gene previously implicated in both T2D and fasting glucose levels by CVASs 53, 54. The melatonin receptor 1B is an inhibitory G protein-coupled receptor (GPCR) that modulates insulin secretion (Figure 2B). It has attracted attention both because of the therapeutic value of GPCRs but also because of the potential link between circadian rhythm and metabolic control; release of melatonin from the pineal gland is inhibited by light, and thus the endocrine hormone is thought to reduce insulin secretion during the night. Because *MTNR1B* expression was initially observed to be higher in individuals with T2D than in controls it was expected that the common risk variant would increase expression [54]. This, in turn, would lead to inappropriately high inhibition of insulin secretion by melatonin. Resequencing of the region, however, identified several rare variants that caused pronounced decreases in receptor function [49]. The variants were too rare to allow association tests at the level of individual variants but were aggregated to increase statistical power, a common practice in RVASs [6]. Grouping based on functional effect established that alleles harbouring rare loss-of-function variants significantly increases susceptibility to T2D (OR = 5.7). By contrast, a meta-allele consisting of all rare variants shows a diluted effect (OR = 3.3). This demonstrates the importance of correctly assigning pathogenicity to exclude incidental mutations and thereby increase power. In addition to discovering an unexpected direction of effect, the study also provides proof-of-principle that rare variants with large effect sizes can be found by resequencing loci prioritized by CVAS.

Targeted resequencing has also been remarkably successful in shedding light on the direction of effect at the *SLC30A8* (solute carrier family 30 member 8) locus [50]. *SLC30A8* encodes a Zn^2+^ transporter, ZnT-8, that localises to the insulin-containing secretory granules (Figure 2B). It was first proposed that a common non-synonymous variant identified by CVAS would decrease function of the transporter, leading to an insulin crystallization defect. Nevertheless, follow-up functional studies and animal models have only reported small, incongruent effects on β cell function 55, 56, 57, 58, 59 (reviewed in [60]). To resolve this, Flannick *et al.* genotyped 150 000 individuals looking for T2D-association of rare variants with large functional effects [50]. By aggregating all protein-truncating mutations, they were able to show at an exome-wide level of significance that loss-of-function variants are protective against T2D (OR = 0.34). Demonstrating the power of human genetics to determine mechanistic directionality, this surprising finding shows that a 50% reduction of ZnT-8 levels decreases the risk of developing T2D by almost threefold. To understand the underlying protective mechanism and evaluate the potential of ZnT-8 as a therapeutic target, these new insights will need to be reconciled with existing data, as discussed elsewhere [60].

Two RVASs so far have attempted to uncover new loci by non-targeted approaches 51, 52. Huyghe *et al.* provided proof-of-principle using an exome-chip approach to investigate several T2D-relevant traits in healthy adults [51]. The study identified associations for insulinogenic index (IGI), a measure of insulin release in response to glucose, and proinsulin (PI) levels, a proxy for β cell stress. The three genes found to be implicated are all strong biological candidates: *PAM* (peptidylglycine α-amidating monooxygenase; associated with IGI) encodes an α-amidase localising to the membrane of secretory granules, and both *KANK1* (KN motif and ankyrin repeat domains 1) and *TBC1D30* (TBC1 domain family, member 3; associated with PI) encode proteins involved in G-protein signalling (Figure 2B).

More recently, the deCODE consortium has studied a cohort of Icelanders to test for associations of rare and low-frequency variants with T2D [52]. By combining WGS of a limited number of individuals with imputation based on Icelandic genealogy they were able to obtain an effective sample size of close to 300 000 individuals. This provided sufficient power to establish four previously unreported association signals in three regions: *PAM*, in which a rare variant had also previously been associated with IGI (see above), *CCND2* (cyclin D2), and *PDX1* (pancreatic and duodenal homeobox 1) (Figure 2B). Interestingly, the intronic variant in *CCND2* was protective against T2D (OR = 0.5) but was associated with higher body mass index (BMI) and height. Cyclin D2 is a widely expressed regulator of the G1/S cell cycle transition and has been shown to influence the proliferative capacity of β cells among many other cell types [61]. *PDX1* encodes a transcription factor important for β cell maturation and is known to be involved in Mendelian forms of diabetes [27]. Accordingly, a rare frameshift mutation was shown to have a moderately high effect (OR = 2.3) [52].

The discovery of *PDX1* as a T2D risk locus expands the overlap between genes implicated in T2D susceptibility and genes known to be involved in Mendelian diabetes (e.g., *KCNJ11, HNF1A, HNF1B, WFS1*). Blurring the boundary between the two diseases, it has recently been shown that the apparent deterministic effect of particular MODY-mutations may reflect an inflated estimate of effect size as a result of ascertainment bias [62]. As more rare variants with large effect sizes become associated with T2D, the operational cut-off point for classification of a mutation as an incompletely penetrant Mendelian variant or a rare T2D mutation with a high effect size will become increasingly arbitrary.

## Concluding remarks and future perspectives

Human genetics has proven a powerful instrument to explore the genetics of β cell dysfunction. Substantial progress has been made from studies into Mendelian forms of diabetes, which have improved our understanding of β cell biology and delivered validated drug targets. Translation of initial T2D variants has been slower, but recent studies have shown progress in elucidating the mechanisms underlying several common and low-frequency susceptibility variants (Figure 2) 37, 51, 52. Several large-scale sequencing efforts by international consortia are currently underway, and these will undoubtedly further expand the number of robustly associated genetic variants. Although it seems unlikely that the first generation of such studies will uncover a large proportion of the missing heritability, they may shed much needed light on the overall distribution of variant frequencies and effect sizes at T2D loci.

One of the greatest challenges in the field remains to bridge the gap between regulatory variants and causative transcripts, but several new methods are promising to accelerate this process. First, the identification of rare, coding variants in regions associated with common regulatory variants can provide prior evidence in favour of particular transcripts. Proof-of-principle RVASs have already been published, but studies with larger sample sizes will provide greater statistical power 51, 52. A second approach is based on annotation of the islet genome through the integration of large-scale genomics and transcriptomics datasets. Understanding the genomic context of genetic associations enables the identification of a functionally active subset of variants and even prediction or direct determination of the impact that specific alleles have on chromatin states. A large number of studies have already contributed to annotating the islet genome, creating an atlas with a growing number of structural and functional layers. As an extension of this principle, *cis*-eQTL studies promise to deliver an additional level of information by identifying correlations between genotypes and expression levels.

Once a causative gene has been inferred, the discovery must be followed up by studies to interrogate the biological function of the encoded gene product. Only then can we gain new insights into β cell biology and appreciate the full implications for disease pathogenesis. For such follow-up studies, traditional characterisation of animal models is being increasingly supplemented by new human model systems. The generation of the first glucose-responsive human β cell line was recently reported [63]. Although still an imperfect model, the cell line represents a major step forward and will complement current work in rodent cell lines. In addition, advances are being made in both the derivation of induced pluripotent stem (iPS) cells and in their subsequent differentiation to the endodermal lineage 64, 65, 66. Soon patient-derived iPS cells will be used routinely to study β cell function. Novel genome-editing tools such as the CRISPR/cas system are already available for fast and simple genetic manipulation of cell lines and iPS cells [67]. In this way it is possible to create both knockouts and models carrying risk alleles with more subtle effects on splicing or function. Finally, appropriate physiological characterisation of human variant-carriers will provide increasingly detailed insight into pathophysiological mechanisms.

Future studies evaluating genetic associations will be supported by such new methods, and thereby accelerate the process of going from variant through transcript to biology. The insights arising from human genetics will continue to improve our understanding of β cell function (Box 4), inspiring the development of novel drugs and facilitating advances in replacement therapies.

Box 4 **Outstanding questions**

• How is the balance between secretion, proliferation, and apoptosis ultimately regulated and disturbed by T2D susceptibility variants that exert their effect through β cells? Translating more variants to biological mechanisms will improve our understanding of this complex system and thereby also diabetes pathogenesis (Figure 3).
• Will larger *cis*-eQTL studies and other functional annotations, such as chromatin conformation capture (3C) (Box 2), deliver the anticipated advance in facilitating translation of regulatory variants to molecular mechanisms?
• Will rare variants contribute significantly to the missing heritability of T2D? In addition, will rare variants, similarly to common variants, primarily exert their effect on diabetes susceptibility through β cell dysfunction?
• What proportion of rare variants will be found in regulatory regions of the genome? There is reason to believe that rare variants with moderate and high effects are less likely to be regulatory than common variants because of the more dramatic effect that coding mutations can have on protein function.

## Glossary

Causative transcript: the transcript through which a genetic variant exerts its effect on disease pathogenesis.
Common variant associations study (CVAS): a type of GWAS targeting variants with a minor allele frequency (MAF) higher than 5%; Box 1 for details.
Expression quantitative trait loci (eQTL): a variant linked to the expression of a specific gene. eQTLs that map near the affected transcript are referred to as *cis*-eQTLs and those that are distal to the transcript are *trans*-eQTLs.
Exocrine pancreatic insufficiency: inadequate levels of digestive enzymes secreted by the exocrine part of the pancreas.
Fine-mapping: the process of refining a disease-associated region by targeted genotyping or sequencing in additional individuals, often from multiple ancestries.
Genetic variant: describes any type of variation at a given genomic position, including single-nucleotide changes and structural variation (deletions and insertions). Genetic variants can be classified based on whether they are coding or non-coding (regulatory) and causative or incidental. Coding mutations can be further divided into non-synonymous and synonymous variations depending on the effect they have on the encoded amino acids. Both non-synonymous mutations and structural variants can be protein-truncating if the translated protein is shorter than the wild type.
Genome-wide association study (GWAS): broad term that encompasses both CVASs and RVASs. Box 1 for details.
Haploinsufficiency: deviation from wild type state due to having only a single functional copy of a given allele.
Mendelian diabetes: covers those forms of diabetes which show a Mendelian pattern of inheritance (also sometimes referred to as monogenic diabetes). The term includes both maturity onset diabetes of the young (MODY) and neonatal diabetes mellitus (NDM), the two most common forms of Mendelian diabetes.
Minor allele frequency (MAF): frequency of the least common allele at a particular locus.
Missing heritability: the gap between the heritability accounted for by currently identified susceptibility variants and the variance in disease susceptibility observed by population genetics.
Odds ratio (OR): used in GWASs as a measure of the effect size for a specific variant. It is defined as the ratio between the proportion of cases and controls carrying a genetic variant.
Penetrance: proportion of mutation carriers that express the expected phenotype. A high penetrance is equivalent to a high OR. A highly penetrant, disease-causing variant will be rare in the general population because of reduced fitness. This trade-off between effect-size and frequency is exploited in the search for low-frequency and rare T2D susceptibility variants which could potentially have a higher OR.
Physiological characterisation: in the context of diabetes susceptibility, physiological characterisation refers to the measurement of several quantitative glycaemic traits. Frequently measured parameters include fasting proinsulin (PI) levels, insulinogenic index (IGI), and various indices of glucose tolerance. Such characterisation can provide insights into whether insulin resistance or β cell dysfunction is driving particular associations.
Rare variant association study (RVAS): a type of GWAS targeting variants with a MAF lower than 5% (low-frequency and rare variants); Box 1 for details.
Stretch enhancer: extended regulatory region bound by multiple transcription factors and exhibiting tissue-specific enhancer activity. Stretch enhancers mirror the related concepts of superenhancers, clusters of open regulatory elements (COREs), and locus control regions.
